# Iron (III)-Quercetin Complex: Synthesis, Physicochemical Characterization, and MRI Cell Tracking toward Potential Applications in Regenerative Medicine

**DOI:** 10.1155/2020/8877862

**Published:** 2020-12-29

**Authors:** Phakorn Papan, Jiraporn Kantapan, Padchanee Sangthong, Puttinan Meepowpan, Nathupakorn Dechsupa

**Affiliations:** ^1^Research Unit of Molecular Imaging Probes and Radiobiology, Department of Radiologic Technology, Faculty of Associated Medical Sciences, Chiang Mai University, Chiang Mai 50200, Thailand; ^2^Department of Chemistry, Faculty of Science, Chiang Mai University, Chiang Mai 50200, Thailand

## Abstract

In cell therapy, contrast agents T1 and T2 are both needed for the labeling and tracking of transplanted stem cells over extended periods of time through magnetic resonance imaging (MRI). Importantly, the metal-quercetin complex via coordination chemistry has been studied extensively for biomedical applications, such as anticancer therapies and imaging probes. Herein, we report on the synthesis, characterization, and labeling of the iron (III)-quercetin complex, “IronQ,” in circulating proangiogenic cells (CACs) and also explore tracking via the use of a clinical 1.5 Tesla (T) MRI scanner. Moreover, IronQ had a paramagnetic T1 positive contrast agent property with a saturation magnetization of 0.155 emu/g at 1.0 T and longitudinal relaxivity (r1) values of 2.29 and 3.70 mM^−1^s^−1^ at 1.5 T for water and human plasma, respectively. Surprisingly, IronQ was able to promote CAC growth in conventional cell culture systems without the addition of specific growth factors. Increasing dosages of IronQ from 0 to 200 *μ*g/mL led to higher CAC uptake, and maximum labeling time was achieved in 10 days. The accumulated IronQ in CACs was measured by two methodologies, an inductively coupled plasma optical emission spectrometry (ICP-EOS) and T1-weighted MRI. In our research, we confirmed that IronQ has excellent dual functions with the use of an imaging probe for MRI. IronQ can also act as a stimulating agent by favoring circulating proangiogenic cell differentiation. Optimistically, IronQ is considered beneficial for alternative labeling and in the tracking of circulation proangiogenic cells and/or other stem cells in applications of cell therapy through noninvasive magnetic resonance imaging in both preclinical and clinical settings.

## 1. Introduction

In the era of cell-based therapy and regenerative medicine, ethical, safe, and efficient treatment protocols are vitally important issues [1–3]. Currently, the magnetic resonance imaging (MRI) of magnetically labeled stem cells represents the only clinically applicable imaging method for highly sensitive, noninvasive, and nonradiation detection protocols. Notably, MRI can be performed serially over time for tracking transplanted stem cells [1, 4, 5]. MRI contrast agents for cell labeling can be used with both T1 and T2 contrast agents, which provide hyperintensity (brightening) and hypointensity (darkening) of T1-weighted and T2-weighted images, respectively. Most magnetic resonance imaging probes of labeled stem cells in preclinical and clinical procedures are based on iron-based contrast agents (IBCAs), while superparamagnetic iron oxide nanoparticles (SPIONs) provide negative T2- or T2^*∗*^-weighted images [6]. Multiple characteristics of the nanoparticles determine the labeling efficacy of the agents, including the size of the iron oxide particle, as well as the shape, charge, and nature of the coating result [7, 8]. These physicochemical characteristics affect not only the efficacy of the particles for MRI but also their stability, biodistribution, metabolism, and degree of clearance [9–13]. Currently, iron (III) is an organometallic ion with biocompatibility, which is well known in terms of its biochemistry and homeostasis in living cells and organisms [14]. It has been developed for both T1-positive [10, 15–17] and T2-negative [18, 19] contrast enhancements. Iron (III) is a d-block transition metal, with five unpaired electrons favoring the formation of six-coordination bonding to the bidentate or tridentate ligands in an octahedral molecular geometrical arrangement [20]. The iron chelators are obtainable by being synthesized or extracted from natural resources for the treatment of iron overload [20, 21] and for imaging probes [15]. To date, dietary flavonoids such as quercetin, rutin, morin, kaempferol, and luteolin have demonstrated potent metal-chelating properties [22–27]. In particular, quercetin has demonstrated strong cardiovascular protection capabilities, anticancer behaviors, and therapeutic activities in both *in vitro* and *in vivo* research [28–30]. However, the poor water solubility, chemical instability, and low bioavailability of quercetin can greatly limit its biomedical applications [31]. Determining the metal-chelating properties of quercetin, it can be seen that quercetin consists of three phenolic rings including A, B, and C rings that are observed in the molecular structure. These rings contain three possible metal-chelating sites that are identified as (1) C3-hydroxy-C4-carbonyl, (2) C4-carbonyl-C5-hydroxy, and (3) the ortho-dihydroxyl (catechol) groups [26]. In addition, both the neutral form (H5QT) and the deprotonated forms (H4QT-, H3QT_2-_, H2QT_3-_, HQT_4-_, and QT_5-_) possess levels of potency to chelate metal ions [32]. The complexation of quercetin and a large number of metal ions has been reported. This indicates that the biological activities of this complex are improved and increased compared to those of free quercetin [33–37].

According to our knowledge, the application of an iron (III)-quercetin complex (termed “IronQ”) is capable of serving dual purposes as T1 imaging probes for MRI and inducing the circulating proangiogenic cells (CACs) that are derived from peripheral blood mononuclear cells (PBMCs). To date, this CAC growth capability has only been established by our research team [38, 39]. Moreover, the IronQ complex enhances radiation-induced cell death in human erythroleukemic cell lines, doxorubicin-resistant leukemic cells (K562/Adr), and their parental cells (K562) by increasing the generation of intracellular reactive oxygen species (ROS) [40]. However, the chemical structure and chemical properties of IronQ have not yet been established or fully investigated. In the present study, we identified the stoichiometry and synthesis methodology of this complex. Furthermore, we characterized the physicochemical properties and MRI properties of the IronQ, as well as the phenotypic features. The angiogenic potential of circulating proangiogenic cells was also investigated via the induction of PBMCs with IronQ. Moreover, IronQ's labeling efficiency into CACs was determined using an inductively coupled plasma optical emission spectrometer (ICP-OES) in parallel with magnetic resonance imaging at 1.5 T.

## 2. Materials and Methods

### 2.1. Materials

Quercetin hydrate, HPLC-grade methanol, and iron (III) chloride were purchased from Sigma-Aldrich (MO, USA). Potassium hexacyanoferate (II) trihydrate was purchased from Merck (Darmstadt, Germany). Roswell Park Memorial Institute (RPMI) 1640 medium and fetal bovine serum (FBS) were obtained from Thermo Fisher Scientific (MA, USA). Endothelial Growth Medium-2 Bullet Kit (EGM-2) and Endothelial Basal Medium-2 (EBM-2) were purchased from Lonza (Basel, Switzerland). All chemicals were of analytical grade. Ultrapure water (specific resistivity of 18.2 MΩ·cm at 25°C) was prepared using a PURELAB Option-Q system (ELGA LabWater; High Wycombe, UK).

### 2.2. Determination of Stoichiometry

The method of continuous variations, or Job's method [41], was used to determine the stoichiometry of the metal-ligand complex. In this method, experiments were conducted to establish the complex between iron (III) and quercetin. The stock solution was freshly prepared in 1 × 10^−3^ M consisting of iron (III) chloride in water and quercetin hydrate in methanol. The quercetin solution was adjusted to a pH of 12 with 1 M NaOH before performing the reaction. These two solutions were combined to a total volume of 10 mL at the following ratios of iron (III):quercetin: 9 : 1, 4 : 1, 3 : 1, 2 : 1, 1.5 : 1, 1 : 1, 1 : 1.5, 1 : 2, 1 : 3, 1 : 4, and 1 : 9. The reaction processes were performed at 25°C for 2 h. The absorption spectra were then measured using an Agilent 8453 UV-visible spectrophotometer (Agilent Technologies; Santa Clara, California, USA). The complex stoichiometry was determined from the graph, in which the level of absorbance at 480 nm and the mole fraction of iron (III) to quercetin were plotted.

### 2.3. Synthesis of the IronQ Complex

Quercetin hydrate (0.0050 mole) was added to 500 mL methanol in round bottles containing an electromagnetic stirrer and a thermometer. The stirred quercetin hydrate was completely dissolved until the color of the solution became yellow. The quercetin hydrate solution was then adjusted to a pH of 12 by slowly adding a 50% (w/v) NaOH solution to change the quercetin from a protonated to a deprotonated form. Iron (III) chloride (0.0025 mole) in 500 mL ultrapure water was freshly prepared and mixed with the deprotonated quercetin solution until the color of the solution changed to dark yellow. The reaction of the combined solution was incubated at 60°C for 2 h under continuous stirring. The combined solution was purified by the dialysis method (MWCO: 12000–14000, Cellu SepT3, USA) and then evaporated to dryness using a rotary evaporator, BUCHI Rotavapor R-100 (BÜCHI Labortechnik AG; Flawil, Switzerland). The dark powder product was then collected and stored in a desiccator at room temperature and kept away from the light.

### 2.4. Characterization and Instruments

The UV-vis absorption spectra of quercetin, deprotonated quercetin, ferric ion, and IronQ were measured in an appropriate solvent using 1 cm quartz cells with a full scan spectrum of 198–1100 nm with an Agilent 8453 spectrophotometer. The IR spectra were determined using the KBr pellet method in a range of 400–4000 cm^−1^ on a Nicolet 6700 FTIR spectrometer (Thermo Scientific; MA, USA). The unique crystalline phase of the complex was studied using a Rigaku SmartLab X-ray diffractometer (Rigaku Corporation; Tokyo, Japan) on a copper anode from 2° to 80° for the diffraction angles (2*θ*). The morphological investigation was conducted using a field emission scanning electron microscope, JEOL model SEM, JSM-5910LV (JEOL; Tokyo, Japan), operating at 15 kV, followed by element analysis via electron-dispersive X-ray spectroscopy (EDS). The particle size of the IronQ was imaged using a transmission electron microscope (TEM), JEOL model JEM-2010, USA, operating at 200 kV. Then, TEM images were taken to measure the particle size with the ImageJ software (viable download at https://imagej.nih.gov/ij/download.html), and the distribution of the particle size was analyzed using the OriginPro 8 software (Northampton, MA, USA). The hydrodynamic size and zeta potential of IronQ were measured at 25°C using folded capillary zeta cells with a Zetasizer Nano ZS (Malvern Panalytical; Malvern, UK). The identification of iron content was confirmed by using a Perkin ELAN-DRCe inductively coupled plasma optical emission spectrometer (Perkin Elmer; MA, USA). The magnetic properties of IronQ were recorded using a vibrating sample magnetometer (VSM).

### 2.5. Isolation of Human Peripheral Blood Mononuclear Cells (PBMCs)

Human peripheral blood mononuclear cells (PBMCs) were obtained from the peripheral blood of healthy human donors using the density gradient centrifugation method [38]. The procedures were approved by the Human Research Ethical Committee of the Faculty of Medicine, Chiang Mai University, Thailand (ref. no. NONE-2560-05052). Firstly, 60 mL of whole blood was collected in heparinized tubes (1430 USP units). Furthermore, 15 mL of whole blood was transferred to a new 50 mL sterile tube, and then, 15 mL of phosphate buffer saline (PBS, pH 7.40) was added. Next, the solutions were gently mixed. Subsequently, 15 mL of Ficoll-hypaque (Lymphoprep™) was carefully injected into the bottom of the tube prior to centrifugation at 1500 rpm for 30 min. The PBMC fraction was then collected from the interphase and washed once with sterile PBS. The cell pellets were then resuspended in a red blood cell (RBC) lysing solution for 5 min. After that, the cells were washed twice with sterile PBS and then resuspended at a density of 1 × 10^6^ cells/mL in an RPMI 1640 medium with L-glutamine supplemented with 10% fetal bovine serum and 1% penicillin/streptomycin (BioMedia, Singapore). The PBMCs were then cultured at 37°C in a humidified atmosphere with 5% CO_2_.

### 2.6. Cell Culture and Treatment for Morphological Observations

Specifically, 1 × 10^6^ cells/mL of the PBMC concentration was seeded in 6-well plates with 4 mL of the RPMI 1640 medium in the presence of 10% FBS and 1% penicillin/streptomycin. For the control group, the PBMCs were cultured in the RPMI 1640 medium only. For the experimental group, PBMCs were treated with an RPMI 1640 medium containing 125 *µ*g/mL of the IronQ complex. PBMCs were incubated at 37°C in a humidified atmosphere with 5% CO_2_ for 14 days. The cell-cultured medium was replaced with 50% fresh completed medium every 3 days. The cell morphology of the PBMCs was observed daily under an ECLIPSE Ts2 inverted microscope (Nikon; Tokyo, Japan), and the results were recorded using the NIS-Element D software (Nikon; Tokyo, Japan).

### 2.7. Peripheral Blood Mononuclear Cell (PBMC) Proliferation Assay

The 3-(4, 5-dimethylthiazol-2yl)-2, 5-diphenyltetrazolium bromide (MTT) colorimetric assay was performed as described previously [42]. Briefly, 1 × 10^6^ PBMCs were suspended in 100 *μ*L of the RPMI 1640 medium in the presence of 10% fetal bovine serum (FBS) and 1% penicillin/streptomycin and seeded in 96-well plates. Then, IronQ at a final concentration of 125 *μ*g/mL was added and further incubated for the indicated time. The IronQ was evaluated at a final concentration of 125 *μ*g/mL following the primary screening. IronQ was previously tested at serial dilutions ranging from 25 to 1000 *μ*g/mL on PBMC cells. Accordingly, 125 *μ*g/mL of IronQ showed the highest differentiation efficiency without causing toxicity over long-term incubation. After the indicated time point, the supernatants containing IronQ were removed to eliminate the influence of IronQ on the absorption spectra of the formazan solution. After that, the cell pellets were collected and resuspended in 80 *μ*L of the RPMI 1640 medium containing 20 *μ*L of the 5 mg/mL solution of MTT, followed by 4 h incubation. The supernatant in each well was then carefully removed, and 100 *μ*L dimethyl sulfoxide (DMSO) was added to dissolve the formazan crystal product in the metabolically viable cells. Absorbance was measured with a BioTekTM EonTM microplate reader (BioTek, USA) at 550 nm.

### 2.8. Phenotypic Characterization of CACs

The IronQ-induced proangiogenic cells were characterized by an immunofluorescent staining assay. Covered glass slides of CACs were fixed with 4% formaldehyde for 10 min and blocked by 5% bovine serum albumin for 1 h at room temperature. The slides of the CAC cells were then incubated with primary antibodies against CD34, CD14, CD133 (Miltenyi Biotec; CA, USA), CD31, CD45, CD105, and VEGFR-2 (eBioscience; CA, USA) overnight at 4°C. This step was followed by rinsing the CACs three times with PBS and incubating them with secondary antibodies for 1 h at room temperature. Finally, the cells were examined under a fluorescence inverted microscope (Nikon; Tokyo, Japan).

### 2.9. Angiogenesis Assay

The angiogenic potential was tested in the presence of a basement membrane matrix using an *in vitro* angiogenesis assay kit (Abcam; MA, USA) according to the manufacturer's instructions. Briefly, an extracellular matrix solution was placed in a 96-well plate at 37°C for 1 h to allow the matrix solution to solidify. For the control group, human umbilical vein endothelial cells (HUVECs; ATCC® CRL-1730™) were seeded onto Matrigel in 5 × 10^4^ cells/well in Dulbecco's modified Eagle's medium with nutrient mixture F-12 (DMEM/F12) supplemented with 20% FBS (Gibco; USA), 0.1 mg/mL heparin (Sigma-Aldrich; MO, USA), 5 ng/mL basic fibroblast growth factor (PeproTech; NJ, USA), and 10 ng/mL epidermal growth factor (Invitrogen; CA, USA) and incubated for 24 h to allow for the formation of tubes. To compare the proangiogenic effects of IronQ-induced CACs on endothelial cells, a Matrigel-based capillary-like tube formation assay was performed using the coculturing HUVECs with CACs in the conditioned medium (CM). The IronQ-induced CACs were mixed with HUVECs at a ratio of 1 : 2, seeded in Matrigel, and then, cocultured in a mixture of the CM and HUVEC growth medium (2 : 1, *v*/*v*). The formed tubules were monitored every 4 h, and cell images were taken with a fluorescence inverted microscope. Cumulative tube length and tube numbers per well were measured using the simple neurite tracer-plugin of Fiji software (http://fiji.sc/Downloads).

### 2.10. MRI Phantom Preparation and Relaxivity Measurements

Phantoms of IronQ at various concentrations of 50, 100, 125, 250, 500, 750, and 1000 *μ*g/mL were prepared to ultrapure water and human plasma at a final volume of 10 mL per tube. Additionally, ferric ions (FeCl_3_) at concentrations of 0.1, 0.2, 0.3, 0.4, and 0.5 mM were used to prepare the phantoms. The iron content of IronQ was measured with an ELAN-DRCe Inductively Coupled Plasma-Optical Emission Spectrometer (ICP-OES) (PerkinElmer; MA, USA) to calculate the degree of relaxivity. To avoid the interference of susceptibility artifacts from the surrounding air during the scans, all sample tubes were immersed in a plastic water container and kept at 25°C. A Philips Ingenia 1.5 T MRI scanner (Philips; Amsterdam, Netherlands) and the dStream HeadSpine coil were used to establish the imaging phantoms. MR images were analyzed using a Philips DICOM viewer R3.0 SP15 (Philips; Amsterdam, Netherlands). The longitudinal relaxation times (T1 values) were measured by imaging the groups of samples simultaneously using an inversion recovery turbo spin echo (IR-TSE) pulse sequence with an echo time (TE) of 40 ms, an echo train length (ETL) of 5, a repetition time (TR) of 1000 ms, an inversion time (TI) of 50–700 ms (interval of 50 ms), an average (NSA) of 2, and a sliced thickness of 3 mm. The transversal relaxation times (T2 values) were measured by imaging the same samples as indicated above using a turbo spin echo (TSE) pulse sequence with a TR of 3000 ms; variable TE values of 50, 75, 100, 125, 150, 200, 250, and 350 ms; an ETL of 16; an NSA of 5; and a sliced thickness of 3 mm.

The T1-longitudinal relaxivity (r1) and T2-transversal relaxivity (r2) of IronQ were evaluated using the linear equation [15] as follows:

(1) $$\begin{array}{c} \frac{1}{T\left(1,2\right)}=\;\frac{1}{T{\left(1,2\right)}_{0}}+\;r\left(1,2\right)\times \left[\text{Iron}Q\right], \end{array}$$

where *T* (1, 2) represents the measured *T*1 or *T*2 time of the solution containing the IronQ and *T* (1, 2)_0_ represents the *T*1 or *T*2 time of the blank matrix (human plasma or DI). The degree of relaxivity was obtained from the slope of the linear fit of the abovementioned equation with a representative unit in mM^−1^s^−1^.

### 2.11. PBMC Labeling and *In Vitro* MR Imaging

Subsequently, 5 × 10^5^ cells/mL in 10 mL of PBMC were cultured in T-25 cm^2^ culture flasks in an RPMI 1640 medium containing IronQ at concentrations of 0, 25, 50, 100, and 200 *μ*g/mL in a humidified CO_2_ incubator at 37°C for 1 and 10 days. After the indicated periods of time, the IronQ-labeled cells and adherent cells were washed three times with PBS to remove any unbounded IronQ from the cells. Labeled PBMCs were harvested via trypsinization (0.25% trypsin, 5 min at 37°C), resuspended in PBS, and counted using a hemocytometer. Next, 5 × 10^6^ cells were transferred to 1.5 mL microcentrifuge tubes and spun down by centrifugation at 7000 rpm for 30 sec for further imaging by MRI. The sample tubes were placed in a plastic rack with dimensions of 18.5 cm × 12.5 cm × 6.5 cm and fixed in a water bath to produce a phantom. This phantom was placed carefully in the center of a dStream HeadSpine coil to perform T1-weighted imaging using a Philips Ingenia 1.5 T MRI scanner. PMBC-labeled cells were imaged using the spin echo sequence (SE) with parameters of TE/TR = 11/525 ms, a flip angle = 69°, a field of view (FOV) = 230 mm, a matrix value = 512 × 512, slice thickness = 3 mm, and NSA = 2. The signal intensity of the sagittal image was measured by employing the region of interest (ROI) technique and using a Philips DICOM viewer R3.0 SP15.

### 2.12. Determination of PBMC Labeling Efficiency by Prussian Blue Assay

To verify the intracellular IronQ uptake of the PBMCs, Prussian blue dye was used to stain the iron component. PBMC (1 × 10^6^ cells/mL, 5 mL) cells in a 6-well plate were incubated with or without 125 *μ*g/mL IronQ for 10 days. After that time, the culture medium containing free IronQ outside the cells was removed, and the PBMCs were washed three times with PBS. Next, the cells were fixed with 4% paraformaldehyde at 37°C in a humidified incubator with 5% CO_2_ for 20 min. After that, a fixative reagent was removed, and 10% potassium ferrocyanide in 6% hydrochloric acid was continuously added at a final volume 1 mL. The cells were then reincubated at 37°C for 30 min. After the indicated time, the cell morphology and positively blue-stained cells were observed under an inverted microscope and imaged.

### 2.13. Intracellular Cellular Content of IronQ

Specifically, 5 × 10^5^ cells/mL in 10 mL of PBMCs were cultured in T-25 cm^2^ culture flasks containing IronQ at concentrations of 0, 25, 50, 100, and 200 *μ*g/mL in a humidified CO_2_ incubator at 37°C for 1 and 10 days. After the indicated periods of time, the IronQ-labeled cells and adherent cells were washed three times with PBS to remove any unbounded IronQ from the cells. Labeled PBMCs were harvested by trypsinization (0.25% trypsin, 5 min at 37°C), resuspended in PBS, and counted using a hemocytometer. The cells were collected by centrifugation at 7000 rpm for 1 min. After that, the cell pellets were resuspended in 5 mL of 20% HNO_3_ solution and incubated at 60°C for 6 h under a fume hood. The samples were then evaluated for their iron contents with ICP-OES. This experiment was conducted in triplicate on each day of the experiment.

### 2.14. Statistical Analysis

Data were expressed as mean ± standard deviation values. Statistical differences were assessed using a one-way ANOVA test followed by Tukey's multicomparison for the two groups in the experiment. Notably, *p* < 0.05 was indicative of any statistical significant differences.

## 3. Results and Discussion

### 3.1. Stoichiometry Determination

**Figure 1:**
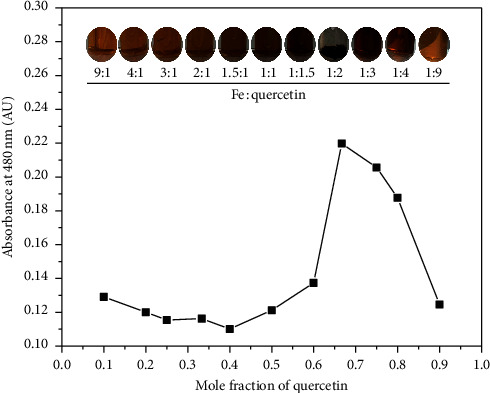
Determination of the stoichiometry of ironQ using Job's method to plot the absorbance at 480 nm and the mole fraction of quercetin. The inset in Job's plot is representative of a photograph of each reaction product.

The method of continuous variation was used to validate the stoichiometric composition of the iron (III)-quercetin complex in a water-methanol solvent system. Iron (III) and quercetin at a concentration of 1 × 10^−3^ M were mixed in equimolar proportions to form the products at different ratios varying from 1 : 9 to 9 : 1. The results are shown in Figure 1. The colors of the chemical reaction products ranged from light brown to dark brown (inset of Figure 1). This corresponded to increasing molar fractions of quercetin as the color turned back to pale brown after passing through a solution with an appropriate ratio of iron (III) and quercetin. The proper ratio of iron (III) and quercetin was indicated at 1 : 2, as this is the ratio at which the desired precipitation of the specified products occurred (brownish-black). This result is similar to that of the spectrophotometric result after investigating the UV-visible absorption band of the complex at 480 nm, which revealed a maximum level of absorbance at 0.667 of the mole fractions for quercetin (Figure 1). The experiment using Job's method indicated a stoichiometric ratio for the reaction between 1 : 2 iron (III) and quercetin in a water-methanol solvent system at an alkaline pH value. Much research indicates that quercetin is a strong chelating agent that can chelate divalent (e.g., Zn (II), Fe (II), Co (II), Ni (II), and Cu (II)) and trivalent (e.g., Fe (III), Sb (III), Ga (III), and Gd (III)) metallic ions at different stoichiometric ratios of 1 : 1, 1 : 2, 1 : 3, 2 : 1, and 2 : 3 for Metal:Quercetin [23–26, 43–47]. Among the metal ions, iron (II) and iron (III) ions favor being chelated by two molecules of quercetin at ratios 1 : 2 and 1 : 1, respectively [43, 48]. However, there are many factors in the experimental conditions that contribute to the formation of a metal-ligand complex, e.g., pH values, oxidizing agents, reactant forms, and solvent systems [49].

### 3.2. Spectroscopic Characterization

**Figure 2:**
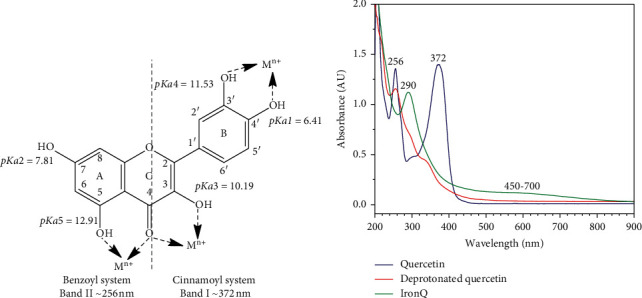
(a) Molecular structure, p*K*a values, and the favored metal-chelating sites of quercetin; (b) UV-visible spectra of quercetin, deprotonated quercetin, and IronQ. Mn^+^ = metal cation with the oxidation state of *n*.

The electronic absorption spectra of the iron (III)-quercetin complex, quercetin, and the deprotonated quercetin were identified using UV-vis spectrophotometry. These recorded spectra are shown in Figure 2(b). The absorption spectra of quercetin in methanol appeared as two major absorption bands at 372 (band I) and 256 (band II) nm, which corresponded to the bands of the cinnamoyl system and benzoyl system, respectively [50]. Quercetin (H5QT) contained five hydroxyl groups (-OH) that were ionizable at different dissociation constant (p*K*a) values (Figure 2(a)). The order of deprotonation was found to be C_4′_-OH, C7-OH, C_3_-OH, C_3′_-OH, and C_5_-OH, with p*K*a1 = 6.41, p*K*a2 = 7.81, p*K*a3 = 10.19, p*K*a4 = 11.53, and p*K*a5 = 12.91, respectively [32, 51]. The titration of quercetin supplemented with NaOH resulted in deprotonated formation (H4QT^−^, H3QT^2−^, H2QT^3−^, HQT^4−^, and QT^5−^), while an increase in the pH values resulted in a bathochromic shift of the electronic absorption spectrum of quercetin. At a pH value of 12, the major deprotonated form of quercetin, HQT^4−^ (deprotonated by 4H^+^), was present at 70%. Consequently, absorbance peaks were observed at 256, 290, and 340 nm. It has been shown that quercetin can be deprotonated by the specific oxidation of the -OH groups at the catechol site with a shift base agent (sodium methoxide) in the presence of free -OH groups at C_3_ on ring C and at C_7_ on ring A, which are known to be responsible for electronic absorption peaks recorded at 348 nm (band I) and 300 nm (band II), respectively [32, 52, 53]. As mentioned above, an absorption peak of 256 nm represents the free -OH group at C_5_ on ring A. Taken together, the occurrence of oxidation of the -OH groups via the addition of NaOH (pH 12 final) is possible at the positions of C_4′_, C_7_, C_3_, and C_3′_ in the quercetin structure (Figure 2(a)). In fact, at the beginning, a pH value of 12 was reached, and an absorbance peak was observed, at 420–425 nm. Subsequently, this peak disappeared over a short period of time as an expected result of the condensation or polymerization between each form of quinones [32, 54]. The absorption peaks of IronQ in water were observed at 290 nm and 450–700 nm. By comparing the absorption spectrum of IronQ to that of the deprotonated quercetin, the peak of 256 nm disappeared (C_5_=O), while the peak of 290 nm (C_7_-OH) for the degree of absorption intensity increased. Notably, a level of absorbance of 420 nm still appeared on the shoulders (Fe–O complex at the positions of C_3_=O and C_4_-OH), and a new absorption broadband at 450–700 nm was observed (Fe–O complex at the catechol sites, C_3′_ = O and C_4′_ = O) [22, 55, 56].

**Figure 3:**
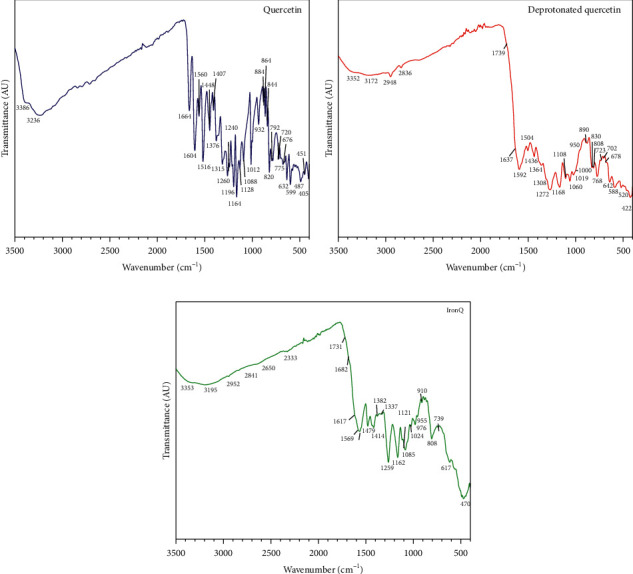
FTIR spectra of (a) quercetin, (b) the deprotonated quercetin, and (c) IronQ.

**Figure 4:**
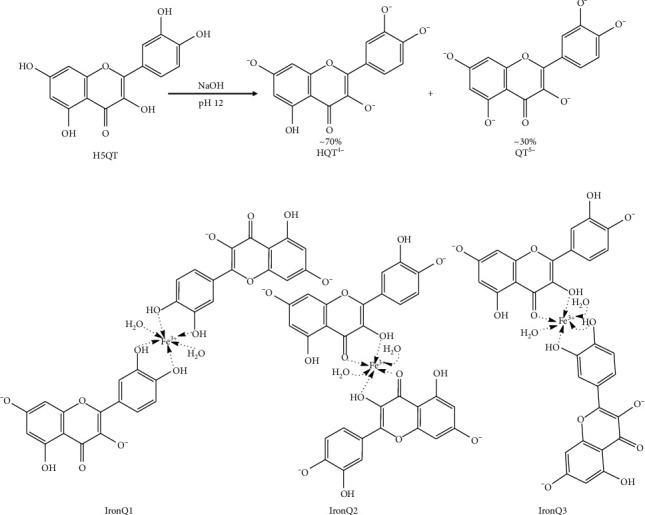
(a) Quercetin deprotonation products observed at a pH value of 12 and (b) the three proposed structures of the iron (III)-quercetin complex.

As stated in the published literature, the UV-vis spectrum of the complex of iron (III) with quercetin can be identified by its characteristic absorption peaks at 290–294, 420–430, and 450–700 nm depending on the environmental conditions and the stoichiometry of the complex formation [55]. The complex of iron (III) with quercetin under acidic conditions of pH 2 in methanol-water revealed major absorption bands at 420 nm and 700 nm, which were ascribed to the formations of 1 : 1 and 2 : 1 iron (III): quercetin complexes, respectively [22, 54]. It was reported that the chemical reaction of iron (III) at the catechol site of gallic acid, gallic acid methyl ester, and catechin resulted in characteristic absorption peaks of 415 nm and 690–695 nm [57], while the chemical reaction of iron (III) at the hydroxychromone site of quercetin revealed an absorption peak of 420 nm [22]. On the other hand, the complex of Gd (III)-quercetin, whose complex site is located at C_4_ on ring C and C_5_ on ring A of quercetin, did not reveal an absorption peak at a wavelength higher than 450 nm [46]. The complex between iron (III) and 3, 5, 7-tri-O-methyl-quercetin (free -OH at the catechol site) revealed a characteristic absorption peak of 600 nm [58]. In this study, the formation of the ferric hydroxide ((Fe (OH)_3_), a typical precipitate formed in an aqueous solution at pH values ranging from 7.0 to 9.0, was not observed [59]. To identify the interaction between the deprotonated quercetin and IronQ, the FTIR spectra, and characteristic bands of pure quercetin, the deprotonated quercetin and IronQ were compared, as shown in Figure 3 and Table 1 [60–62]. We observed that the aryl ketonic stretching C_4_=O at 1664 cm^−1^ of quercetin disappeared, while new strong C=C stretching bands at 1592 cm^−1^ and 1569 cm^−1^ appeared for deprotonated quercetin and IronQ, respectively [52, 53]. The results suggest that as the deprotonation of C_3_-OH occurred, an intrastructure rearrangement resulted in, at least, two resonance aryl ketones (OC_3_=C_4_O^−^) in their molecules being affected by the loss of the C_4_=O stretching signal. Indeed, the delocalization of electrons clearly occurred on rings A, B, and C of the deprotonated quercetin. The deprotonation of the hydroxyl groups of quercetin reflected a loss of C–O–H stretching at 1196 cm^−1^, and the regression signal of O–H bending of the phenols was observed. On the other hand, C–O stretching in the phenol was not observed in the deprotonated quercetin or IronQ. The presence of C–O–C stretching was found in all three samples, while quercetin, the deprotonated quercetin, and iron (III)–quercetin demonstrated that the basic structure of quercetin was not broken. For IronQ, only one new band at 470 cm^−1^ appeared and was ascribed to Fe–O stretching for the Fe (III)–quercetin complex, but in the case of the Fe (II)–quercetin complex, Fe–O stretching was found at a higher wavenumber shift than 630 cm^−1^ [48, 63]. Notably, this confirmed the formation of the iron (III)–quercetin complex. Together with the UV-visible, NMR, and FTIR results, two sites at C_3′_-O-/C_4′_-O- on ring B and C_3_-O-/C_4_=O on ring C of the deprotonated quercetin molecules (HQT^4−^ and QT^5−^) (Figure 4(a)) were involved in the formation of IronQ (as loss of their stretching signals) with a stoichiometry ratio of 1 : 2 for iron (III) and quercetin, respectively. Oxygen atoms at these sites acted as electron donors to form coordinated covalent bonding with iron (III), and two molecules of water (*q* = 2) were also involved in the hexa-coordinated complex [20]. The proposed structures of IronQ at an adjusted pH value of 7.40 are indicated in Figure 4(b). The hydration molecules (*q*) of the metal core (*i*.*e*., Gd^3+^, Mn^2+^, and Fe^3+^) directly resulted in the relaxivity of the contrast agent, while the conventional Gd^3+^-based contrast agents presented only one hydration molecule (*q* = 1) [64, 65]. The two hydration molecules of IronQ involving Fe^3+^ induced a dipole-dipole interaction, leading to a shortening of T1, which resulted in a contrast enhancement on the T1-weighted images [66].

**Figure 5:**
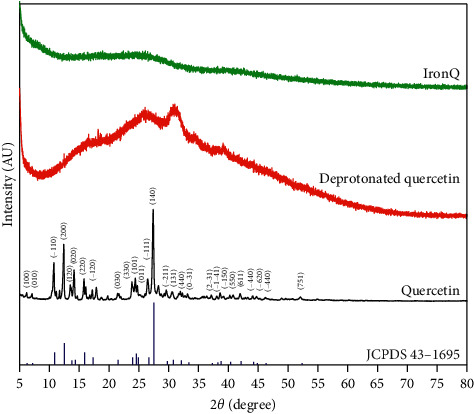
Powder XRD patterns of quercetin, the deprotonated quercetin, IronQ, and the standard quercetin (JCPDS Card No. 43–1685).

The ^1^H-NMR spectra were recorded for quercetin, the deprotonated quercetin, and IronQ at 500 MHz (See Figures S1–S3 in the Supplementary Materials). In the case of IronQ, the NMR profile displayed a similar pattern to the deprotonated quercetin, but broad-spectrum and low-level signals were also detected. In addition, two new multiplets (at *δ* = 2.37 ppm and *δ* = 2.77 ppm) were observed. X-ray powder diffraction (XRD) was used for unique phase determination of the IronQ, and information was provided on the crystalline or amorphous material. The chemical compositions of quercetin, quercetin quinone, and IronQ were established by XRD patterns from 2° to 80°, as shown in Figure 5. The diffractograms of quercetin as quercetin dihydrate revealed a series of intense peaks that were representative of the physicochemical properties [67], while quercetin quinone tended to display an amorphous pattern. Furthermore, the results present a broad XRD pattern for IronQ with no diffraction peak, indicating an amorphous nature.

### 3.3. Particle Morphology and Elemental Analysis

**Figure 6:**
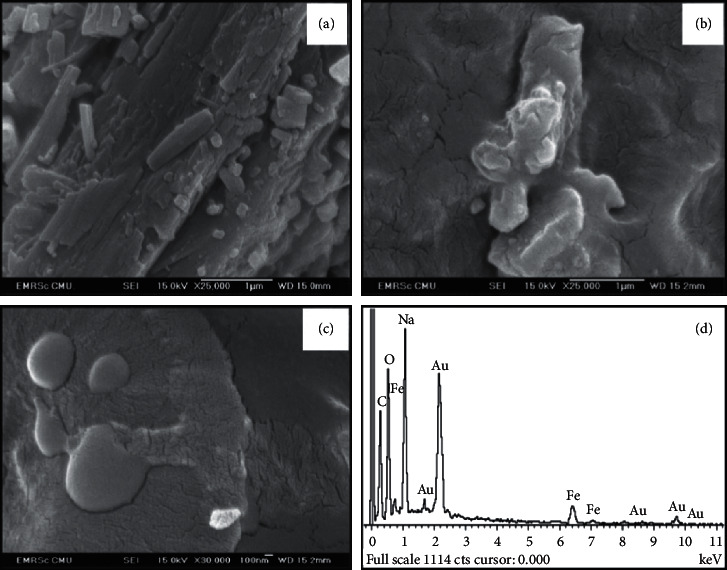
SEM images for the analysis of (a) quercetin and (b-c) IronQ at (b) 25000X and (c) 30000X amplifications followed by (d) the EDX spectrum.

The particle morphology of IronQ was observed using a field emission scanning electron microscope (SEM). The SEM images of quercetin and IronQ were used to evaluate the geometrical shape parameters of the particles. The SEM images of quercetin displayed rod-like or needle-like shapes (Figure 6(a)). In contrast, IronQ showed a smooth surface and an amorphous nature (Figures 6(b) and 6(c)). The element profiles on the surface of the IronQ were analyzed by energy-dispersive X-ray (EDX) spectroscopy. The EDX spectrum of the IronQ, as shown in Figure 6(d), revealed that the compositions of C, O, Na, and Fe were 46.57%, 37.29%, 13.07%, and 3.07%, respectively. A Na atom was introduced by NaOH titration and might ionically bond with the IronQ at free -C–O- moieties of deprotonated quercetin. This atom was also found to stabilize the complex, as shown in the sodium salt formula. These sodium ions are involved in making ionic bonding to the four deprotonated (C–O^−^) positions on the IronQ structure, as shown in Figure 4(b).

### 3.4. Particle Size and Zeta Potential Analysis

**Figure 7:**
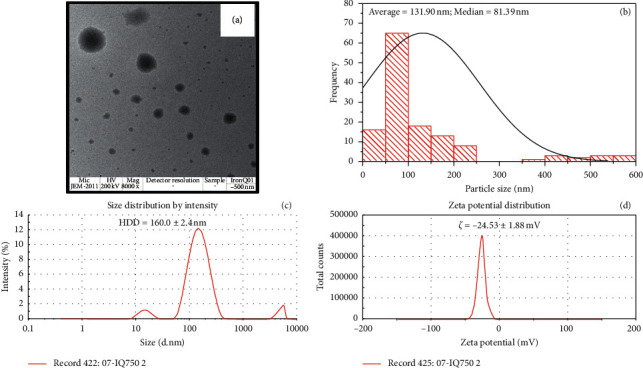
Size and zeta potential of IronQ. (a) TEM image, (b) TEM size distribution (*n* = 132), (c) hydrodynamic size (*n* = 3), and (d) zeta potential of IronQ (*n* = 3).

The morphology and particle sizes of IronQ were characterized using a transmission electron microscope (TEM). The TEM images of IronQ showed spherical shapes of various sizes. The distribution of their sizes ranged from 37 to 600 nm, and the mean and median sizes of the particles were 131.92 and 81.39 nm, respectively (Figures 7(a) and 7(b)). However, the aggregation of the complex was found with a larger spherical shape and size approaching ∼600 nm, while the hydrodynamic diameter (HDD) of the IronQ in ultrapure water, which was adjusted to a pH of 7.4 at 25°C using the dynamic light scattering (DLS) technique, was observed with an average size of 160.0 ± 2.4 nm (Figure 7(c)). In addition, IronQ displayed a negative charge on its surface with the mean zeta potential of −24.53 ± 1.88 mV (Figure 7(d)). The zeta potential value less than −30 mV indicates that IronQ had moderate stability in water; thus, agglomerations could be observed, as shown in Figure 7(d) [68]. The negative zeta potential values indicate that the surface of IronQ has anionic charges; this property generally improves the blood circulation half life of the complex through electrostatic repulsive force with plasma proteins. This outcome reduced the level of protein adsorption and phagocytosis by the reticuloendothelial system (RES) [7, 69, 70].

### 3.5. Magnetic Properties and Phantom MRI Analysis

**Figure 8:**
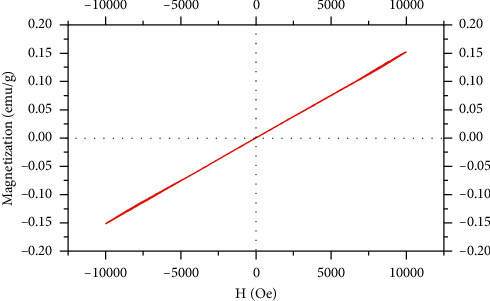
M-H curve of IronQ.

The magnetic property of iron (III)-quercetin was determined by the saturation magnetization (Ms) obtained from the M-H curve using VSM, as indicated in Figure 8. The obtained value was found to be 0.155 emu/g at 1.0 T (10 kOe), indicating that this complex has paramagnetic properties. The lower-level values of the saturation magnetization of IronQ were responsible as a T1-positive contrast (bright image) agent for magnetic resonance imaging (MRI). T1-positive contrast agents, such as gadopentetate dimeglumine (Magnevist®), revealed Ms values equal to 0.397 emu/g at 1.2 T (12 kOe), while the Gd (III)-quercetin complex revealed Ms values equal to 0.405 emu/g at 1.5 T, and the iron (III)-doped calcium phosphate nanoparticles revealed Ms values that were lower than 0.15 emu/g at 1.5 T [71–73]. Notably, the higher levels (>20 emu/g) of the contrast agents, particularly superparamagnetic iron oxide nanoparticles (SPIONs), were observed as a superparamagnetic property and exhibited T2 negative (contrast dark image) in MR imaging [71, 74]. However, the ultrasmall iron oxide nanoparticles (<6 nm) displayed the properties of the T1-contrast agent [10, 75]. Indeed, both the T1 (spin-lattice interaction) and T2 (spin-spin interaction) relaxations of the contrast agents were dependent upon the saturation magnetization of the nanoparticles, their magnetic interactions with the protons of the surrounding water molecules, and their magnetic strength.

**Figure 9:**
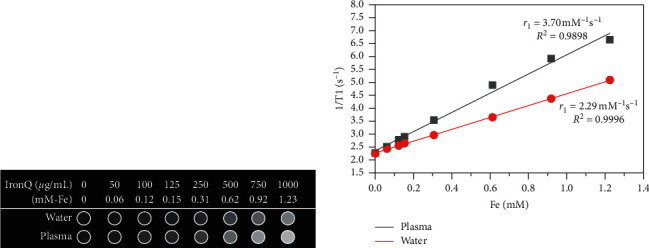
Magnetic resonance imaging properties of IronQ in water and plasma phantoms. (a) T1-weighted image showing different contrasts with increasing concentrations of IronQ (0–1000 *µ*g/mL) and (b) a plot of the relaxation rate 1/T1 versus the IronQ concentration.

The paramagnetic properties of IronQ in the case study may be related to the five unpaired electrons found in the 3d orbit of iron (III) (high spin, *S* = 5/2), leading to an apparent effect identified as spin-lattice interactions under longitudinal relaxation at the applied magnetic field [73]. However, the oxidative state of iron (II) without the magnetic property did not affect T1 relaxation. Numerous iron (III) complexes with small molecules beyond quercetin were representative of a T1-positive contrast agent, such as + Fe-DTPA, Fe-tCDTA, Fe-HBED, and Fe-3FCAT_3_ [15–17]. In general, in the experimental settings for determining the T1 relaxivity of the contrast agent, solvents such as water are most often used, even though they do not mimic the relevant physiological conditions; a physiological medium of human plasma was also used. [76]. T1-weighted images of IronQ's effects were achieved by increasing the image intensity of the water and human plasma in a dose-dependent manner, as indicated in Figure 9(a). Within the range of concentrations of 50–1000 *μ*g/mL (0.06 to 1.23 mM Fe by ICP-OES), this complex revealed strong spin-lattice interactions that corresponded to the higher values of the longitudinal relaxivity rate (1/T1) and the image contrast of the plasma when compared to the water phantom. In addition, the T1 relaxation rate and the cross-relaxation rate were found to be constant between the water, while the plasma proteins were correlated with an increasing percentage of protein contents [76]. The property of IronQ as a positive contrast agent in magnetic resonance imaging was determined from the T1 relaxivity (r1) value that was obtained from the slope of linear fitting as a series of experimental data contrasting between the relaxation rate and the concentration of IronQ, as shown in Figure 9(b) [65]. The longitudinal relaxivity of IronQ was 2.29 mM^−1^s^−1^ for water and 3.70 mM^−1^s^−1^ for the human plasma obtained at 25°C under a magnetic field of 1.5 T. As mentioned above, the T1 relaxivity of IronQ in plasma exhibited a higher value than that in the water phantom. However, we observed the opposite phenomena for ferric ions (Fe^3+^) through the iron (III)-water complex, which revealed a higher degree of longitudinal relaxivity in water than in plasma (r1; water/plasma = 3.70/0.28 mM^−1^s^−1^). In addition, the iron (III)-water complex exhibited a stronger T2 effect in water than in plasma (r2; water/plasma = 17.96/0.42 mM^−1^s^−1^). A reduction in the r1 value of the iron (III)-water complex in plasma might occur as a result of two specific factors: (1) the water-protein ligand exchanges known as ligand effects and (2) the reduction of iron (III) under a high spin to iron (II) oxidation state, identified as a low spin by plasma proteins. Three mechanisms are considered to contribute to the relaxivity of IronQ: (1) inner-sphere relaxation through iron (III)-coordinated water molecule exchanges with other water molecules, (2) second-sphere relaxation where hydrogen bound water molecules are present in the second coordination sphere or an exchangeable hydrogen atom (such as O–H and N–H) between water and plasma proteins that undergoes relaxation and exchange, and (3) outer-sphere relaxation, where water molecules can be diffused close to the IronQ and can also be relaxed [17, 64]. The T2 relaxivity of IronQ was not evaluated in terms of its value because this complex was not involved with the spin-spin relaxation process. The *r*2/*r*1 ratio value was close to zero. This indicates that IronQ acts as a potent T1 contrast agent. In general, T1 contrast agents have a lower *r*2/*r*1 ratio (*r*2/*r*1 < 2), while T2 contrast agents have a larger *r*2/*r*1 ratio (>10) [75]. Through comparison of the relaxivity of IronQ with other iron (III) complexes derived by various kinds of small molecule chelators, IronQ displayed *r*1 values that were higher than those of Fe-DTPA (*r*1; water/serum = 0.6/0.9 mM^−1^s^−1^ at 0.94 T), Fe-tCDTA (r1; water/serum = 2.0/2.2 mM^−1^s^−1^ at 0.94 T), and Fe-HBED (*r*1; water = 0.49 mM^−1^s^−1^ at 1.5 T) but lower *r*1 values than Fe-3FCAT_3_ (*r*1; water = 3.2–7.3 mM^−1^s^−1^ at 4.7 T) [15–17]. Moreover, the same *r*1 values were recorded for ligands such as quercetin, which were different for metal (Gd^3+^). This difference occurs because IronQ is associated with greater *r*1 values than those of the Gd (III)-quercetin complex [72]. Surprisingly, our results showed that the T1 relaxivity value of IronQ was within the T1 relaxivity range of the clinical T1-positive contrast agents, including Gd-DOTA, Ga-HPDO3A, Gd-DO3A-butrol, Gd-DTPA, Gd-DTPA-MBA, Gd-DTPA-BMEA, Gd-BOPTA, and Mn-DPDP, both in water (range of 1.5–4.2 mM^−1^s^−1^) and in plasma (range of 3.4–6.6 mM^−1^s^−1^), which were measured at 37°C and 1.5 T [77].

### 3.6. Proliferation and Morphological Observations of the PBMC-Treated Iron (III)-Quercetin Complex

**Figure 10:**
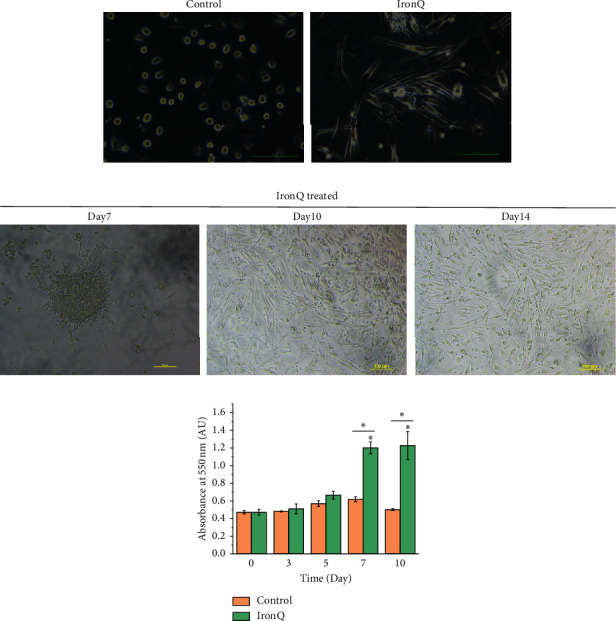
Proliferation and morphological observations on the peripheral blood mononuclear cells- (PBMCs-) treated IronQ complex. (a) Representative phase-contrast images of the morphological states of PBMCs on day 14 of the culturing process. (b) PBMCs were cultured in a culture medium without and with 125 *μ*g/mL of the IronQ complex. CFU-Hill or early outgrowth EPCs were observed after 7 days of culturing, while the circulation of angiogenic cell (CAC) progeny was observed after 14 days of culturing under IronQ conditions. Scale bar = 100 *μ*m. (c) A proliferation assay over 10 days of culturing revealed that the cells generated under IronQ conditions showed greater proliferation (*n* = 3; ^*∗*^*p* < 0.05).

Human peripheral blood mononuclear cells (PBMCs) were cultured in an RPMI 1640 medium with 10% FBS and 1% penicillin/streptomycin without adding any specific growth factors, either in the absence (control) or presence of 125 *μ*g/mL of the iron (III)-quercetin complex (IronQ). The adherent peripheral blood mononucleated cell population appeared under both conditions with different characteristic features. Under IronQ conditions, the cells appeared as long spindle-shaped cells that were of considerable length (∼100 *μ*m), while the majority of cells in the control media appeared shorter in comparison (Figure 10(a)). Morphological observations at different time points revealed that, under IronQ conditions and after continuous culturing for 7–10 days, the cells became confluent and formed colonies. They then displayed a central cluster of rounded and flat cells with a radial arrangement of spindle-shaped cells (Figure 10(b)). These colonies are referred to as the colony-forming unit-Hill (CFU-Hill), as described previously by Hill et al., and were consistent with the endothelial progenitor cell (EPC) phenotype [78]. On day 14, adherent cells appeared homogenously as long spindle cells (Figure 10(b)). In contrast, most cells in the control culture appeared as adherent cells with several shapes and were shorter in comparison. It was revealed that IronQ promoted a greater number of adherent cells with almost 90% confluence on day 14. Furthermore, the IronQ complex treated with PBMCs resulted in an almost 3-fold higher number of cells than that in the untreated controls at the end of the culture period. The quantification of cell proliferation over the 10 days of culturing revealed that the cells generated under IronQ conditions experienced greater proliferation (Figure 10(c)). It is well established that the cells of PBMC consist of terminally differentiated cells, including monocytes and lymphocytes. Along with mature cells, PBMC also contained a very low subset (0.01%) of circulating endothelial progenitor cells [79]. Because monocytes and lymphocytes do not divide without the activated cytokines, under normal culture conditions, the cells will die over time [80]. Surprisingly, culturing PBMCs under IronQ composed of iron (III) and quercetin promoted the proliferation and differentiation of circulating proangiogenic cells (CACs). It has been reported in many studies that quercetin, a natural flavonoid, plays a crucial role in improving endothelial dysfunction [81, 82]. Similar to our findings, a recent study reported that a quercetin-rich-derived onion extract improves endothelial dysfunction and the EPC number in healthy overweight and obese persons [83].

**Figure 11:**
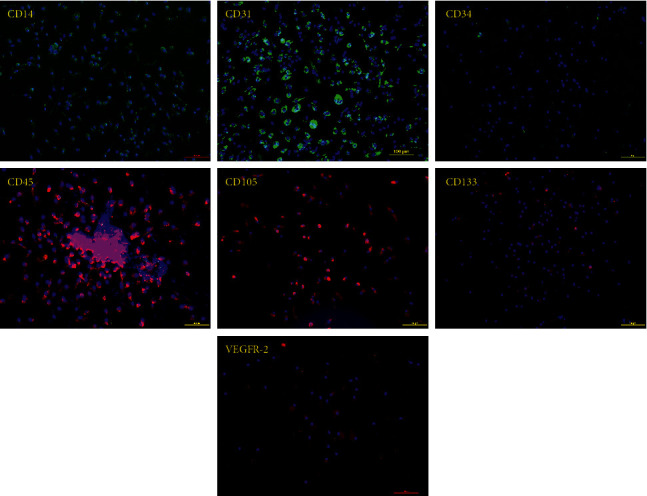
Immunofluorescence staining of IronQ-induced CACs. CACs were stained positively for monocyte and endothelial progenitor cell markers, CD14, CD31, CD45, CD105, CD133, and VEGFR-2 and negatively stained for CD34. Scale bar = 100 *µ*m.

The expanded spindle-shaped cells were further characterized by immunofluorescence staining for the cell surface markers of the stem/progenitor, endothelial cells, and monocytes/macrophages. Immunofluorescent staining showed that the expanded spindle-shaped cells were positive for CD14, CD31, CD45, CD105, CD133, and VEGFR-2 and negative for CD34 (Figure 11). These findings were consistent with the reported characterizations of early EPCs and circulating angiogenic cells [84]. Conversely, a recent study showed that normal PBMCs were negatively stained for EPC markers, including CD14, CD105, VEGFR-2, and CD133, but with a strongly positive expression for the pan leukocyte marker, CD45 [85]. Originally, EPCs were recognized as hematopoietic cells unable to directly form tube-like structures. In contrast, EPCs were able to increase angiogenesis in a paracrine manner and played a role in promoting and modulating angiogenesis [86–88]. In this study, we describe a novel method for generating spindle-shaped fibroblast-like cells from nonmobilized PBMCs using a simplified culture method without any cytokine cocktail additive. Using our home-synthesized MR contrast agent “Iron (III)-quercetin complex,” PBMCs cultured with the IronQ complex displayed a degree of differentiation of cells into spindle-shaped cells with fibroblastic characteristics and enhanced proliferation of blood progenitor cells. Several studies have reported on the differentiation of peripheral mononuclear cells into ﬁbroblast-like cells, which are termed fibrocytes and endothelial progenitor cells (EPCs) or circulating angiogenic cells (CACs) and pericytes, both of which are known to play roles in proangiogenic interactions in both *in vitro* and *in vivo* experiments [89–93]. The results indicate that the cultivation of PBMCs from peripheral blood under the iron (III)-quercetin complex gives rise to proangiogenic progenitor cells. These progenitors, which we identified as circulating proangiogenic cells (CACs), are attractive as an alternative to bone-marrow-derived mesenchymal stem cells or progenitor cells for revascularising tissue after trauma and/or chronic damage. There is mounting evidence for the promising therapeutic properties of transplanted bone marrow-derived or peripheral blood-derived EPCs in the revascularization of ischemic tissue in both experimental and clinical studies [94–96]. To simplify the culture method for expanded EPCs from the PBMC population, we describe here a modified culture method to generate CACs with ease. The ease by which the sources of cells can be noninvasively obtained can reduce the cost of cell preparation. Moreover, by using IronQ to generate CACs for application in cell transplantation, we can reduce the cell tracking step because magnetic resonance imaging can track IronQ.

### 3.7. Proangiogenic Potential of CACs to Promote Tube-like Formation *In Vitro*

**Figure 12:**
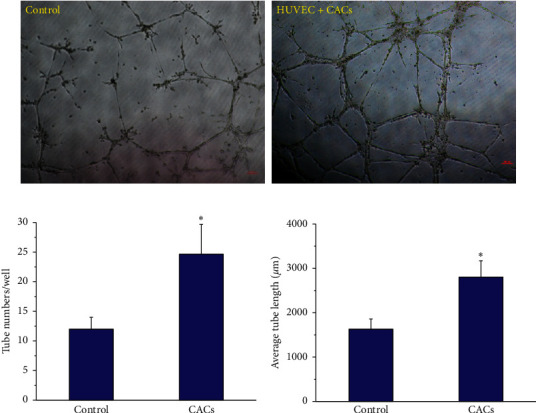
IronQ-induced CACs promoted tube-like formations during coculturing with HUVECs. (a) Representative endothelial tube formation following 24 h of incubation with CACs and their conditioned medium in Matrigel®. Control: HUVEC in the growth medium, HUVEC + CACs: HUVEC coculture with CACs and the condition medium of CACs. Scale bars = 100 *μ*m. (b), (c) Graph showing the average endothelial tube number and tube length (*μ*m). Notably, this assay was performed 8 independent times on matched samples from 8 different donors (^*∗*^*p* < 0.05).

Next, we investigated whether CACs can exert their roles in proangiogenic properties by coculturing them with HUVECs in a semisolid medium Matrigel™ assay. HUVECs were seeded in a growth medium as the control group (Figure 12(a)). Interestingly, within the cocultured system in the presence of CACs, HUVECs increased both the stability of the capillary-like tubes in the semisolid medium and their organizational efficiency. Furthermore, CACs and their conditioned medium induced a pronounced increase in their ability to promote the amount of elongation and tubular networks compared to the control situation (Figure 12(a)). The quantification analysis of the HUVEC tube length and tube number (Figures 12(b) and 12(c)) indicated that CACs and their condition medium significantly increased HUVEC tubulogenesis by approximately two-fold (*p* < 0.05). This characteristic feature was primarily attributable to enhanced angiogenic capacity from the production of angiogenic factors by culturing PBMCs under IronQ complex conditions. Similarly, blood-derived angiogenic cells were previously found to be able to increase and stabilize endothelial tubular structures following direct coculturing with HUVECs, indicating a proangiogenic effect of blood-derived angiogenic cells [91, 92]. A number of studies have also reported the proangiogenic effect of mesenchymal stem cells (MSCs), wherein MSCs derived from human bone marrow cells had the ability to regulate new blood vessel formation, stability, and function [93, 97, 98]. In summary, the data suggest a proangiogenic effect of the expanded circulating proangiogenic cells that were collected from the cultivation of peripheral blood mononuclear cells under the conditions of the MR contrast agent, the iron (III)-quercetin complex.

### 3.8. Intracellular Iron Uptake by Prussian Blue Assay and ICP-OES Analysis

**Figure 13:**
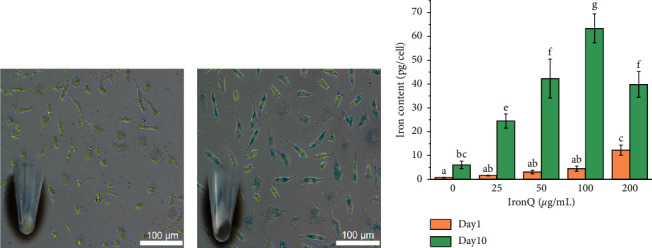
Intracellular iron contents: (a) Prussian blue staining of iron in the nonlabeled PBMCs (control) and (b) 125 *μ*g/mL labeled IronQ. (c) Quantitative analysis of intracellular iron contents of PBMC labeled with 25, 50, 100, and 200 *μ*g/mL of IronQ using ICP-OES analysis for day 1 and day 10. Different lowercase letters indicate significant difference according to the ANOVA analysis followed by Tukey's test (*n* = 3; *p* < 0.05).

An investigation of IronQ inside the cells as a key factor in the uptake rate and internalization dynamics could provide a better understanding of how the intracellular accumulation of this complex can lead to broader applications in imaging probes for MRI, cell labeling, and tracking for stem cell biology and therapy. The accumulation of IronQ in PBMCs was monitored by a Prussian blue assay based on the reaction between the ferric ions and potassium ferrocyanide to produce the formation of blue particles that could be observed under a microscope. We observed that most of the labeled cells under a long exposure time (10 days with 125 *μ*g/mL) were positively stained with a blue color in the cytoplasm of the cells, as indicated in Figure 13(b), and clearly differed from the nonlabeled cells (Figure 13(a)). The brown color of the pelleted cells is depicted in the inset of Figure 13(b), while the nonlabeled cells displayed a pale-yellow color (inset of Figure 13(a)). The amount of IronQ accumulated in the cells was measured by ICP-OES techniques, and the results are shown in Figure 13(c). The accumulation of the IronQ in PBMCs was evident in a dose- and time-dependent manner [9, 99]. PBMCs were exposed to IronQ at concentrations ranging from 0 to 200 *μ*g/mL for a period of 10 days, while the iron content was significantly higher than that of the cells for a shorter period of time involving 1 day of exposure to this complex. We found that the iron contents on day 10 were around 13- to 15-fold higher than those on day 1, which was determined by concentrations of 25 to 100 *μ*g/mL. The highest degree of iron uptake was found at 63.29 ± 6.03 pg Fe per cell in PBMC treated with 100 *μ*g/mL of IronQ for 10 days. For iron content, labeling of the endothelial progenitor cells (EPCs) derived from the PBMCs via SPIO at 20 *μ*g/mL for 24 h detected values of 13.6 ± 1.8 pg Fe per cell [100], which were higher than the values determined by labeling the PBMCs via the T1-contrast agent IronQ (1.65 ± 0.11 pg Fe per cell) at 25 *μ*g/mL for 24 h. However, at the same time of incubation at a higher concentration (200 *μ*g/mL), the PBMCs utilized IronQ at up to 12.31 ± 2.10 pg Fe per cell. In addition, the highly efficient labeling of human mesenchymal stem cells with 25 *μ*g/mL of Citrated SPIONs for 24 h without the use of a transfection agent was reported at 69.6 ± 5.1 pg Fe per cell, while a higher degree of efficiency than that for Endorem SPIONs (25.7 ± 6.5 pg Fe per cell) was observed under the same conditions [9]. However, the iron content decreased from the highest value when the concentration of the IronQ was higher than 100 *μ*g/mL. These contradictory results could be due to the proliferation effect of IronQ. This result confirmed that proliferation occurred in the PBMC-treated IronQ. Thus, as the cells proliferated, the amount of IronQ in each cell decreased, and IronQ uptake could not be detected in some cells [101]. Another reason might be that the maximum iron content achieved for the activities of PBMCs induced by IronQ was ∼70 pg Fe per cell, while the iron overload might have been removed via exocytosis. However, a further study should be conducted to confirm this outcome. Indeed, cell labeling efficiency is dependent upon many factors, including the type of cells (e.g., human endothelial progenitor cells, human mesenchymal stem cells, human embryonic stem cells, human adult neural stem cells, or PBMCs) and cell origin (human or murine); the physicochemical properties (e.g., size, charge, and formulation) of the imaging probes; the concentration values of the probes that had no effect on the viability, differentiation, and function of the labeled cells; and the conditions associated with labeling (e.g., incubation time, cell density, and with or without the aid of transfection techniques) [4, 6, 9, 102–107]. Most of the magnetic resonance imaging probes used to label the stem cells in both clinical and preclinical procedures were based on superparamagnetic iron oxide nanoparticles (SPIONs) that provided negative T2- or T2^*∗*^-weighted images, such as dextran-coated iron oxide (Endorem/Feridex®; the U.S. Food and Drug Administration (FDA) approved it, but the product has been discontinued), carboxymethyl dextran-coated ferucarbotran (Resovist®, which was FDA approved but discontinued), ferumoxytol/Feraheme (an FDA-approved product for iron deficiency treatment in anemia), and Molday ION EverGreen, FeraTrack, and Dargon Green-encapsulated magnetic polymers. These agents under development have been reported to be capable of achieving biocompatible intracellular stem cell labeling, along with the uptake of 2–9 pg Fe per cell [6]. More recently, the labeling of MSCs involved in cellular uptake was proven for more than 10 pg Fe per cell without the use of transfection agents, as reported for very small iron oxide (VSOP; 21 pg Fe per cell) and multicore carboxymethyl dextran-coated iron oxide nanoparticles (multicore particles, MCP; 17 pg Fe per cell) with 2 mM of the imaging probes for 24 h [6]. A higher degree of uptake of the imaging probes by cells could improve the MR imaging signal for hematopoietic progenitor/stem cell tracking in both preclinical and clinical therapies [4, 103, 104, 107].

### 3.9. *In Vitro* Evaluation of Cell Labeling by MRI

**Figure 14:**
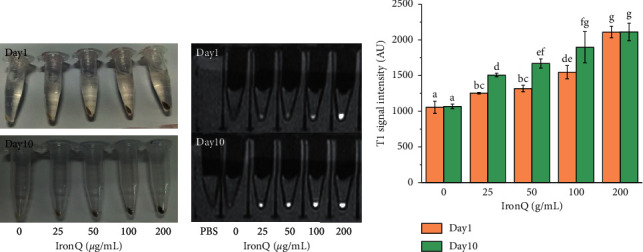
*In vitro* MRI studies: (a) physical observations, (b) T1-weighted images from a sagittal view, and (c) a plot of the T1 signal intensity of PBMC pellets labeled with IronQ at various concentrations in microcentrifuge tubes with phosphate buffer saline (PBS) on day 1 and day 10 of the incubation period. Different lowercase letters indicate significant difference according to the ANOVA analysis followed by Tukey's test (*n* = 3; *p* < 0.05).

As mentioned above, the PBMCs labeled with IronQ were brown colored, and the color gradually changed to dark brown (Figure 14(a)), which corresponded with an increasing concentration of the complex accumulated within the cells (Figure 14(c)). Similar results were observed after the *in vitro* MRI experiments of PBMCs, wherein IronQ enhanced the brightness intensity of T1-weighted images as an indication of the concentration amount (0, 25, 50, 100, and 200 *μ*g/mL) and the incubation time (1 day and 10 days), as shown in Figures 14(b) and 14(c). The highest concentration used in this study, 200 *μ*g/mL, revealed no differences between the maximum T1 signal intensities at day 10 and day 1 of the incubation period (Figure 14(c)). There are many possibilities for this result: (1) During the culture period of PBMCs with IronQ, the useful cells of IronQ in proliferation and differentiation processes and the interactions between IronQ and biomolecules or metabolites in the cells may have resulted in the conformation and/or oxidation state of IronQ changes. These may include IronQ metabolite products, the self-aggregation of IronQ, and the high-spin Iron (III)-quercetin complex changing to a low-spin Iron (II)-quercetin complex involved in reducing T1 relaxivity [108]. Thus, a stability test and pharmacokinetics study of IronQ are necessary to achieve the next goal of improving labeling efficiency and MRI tracking. (2) An unsuitably long repetition time (TR = 525 ms) was used, which did not allow discrimination of the T1 signal intensity under this spin-echo pulse sequence [109]. This result suggests that the appropriate conditions for the labeling of PBMCs and imaging via T1-weighted MRI included 5 × 10^6^ cells, a concentration of IronQ at 200 *μ*g/mL, and 24 h of incubation time at 37°C in a humidified incubator. Moreover, by using the spin-echo pulse sequence, the suitable TR value was less than 525 ms for the IronQ-labeled cell tracking under MRI. However, experimental settings that include a pulse sequence for optimizing IronQ-labeled cell tracking both *in vitro* and *in vivo* are necessary considerations. Indeed, we could label the PBMCs with a higher concentration of IronQ, such as 500 *μ*g/mL, and culture the IronQ labeled cells for a longer period of time at 14 and 21 days, to expand the progeny's angiogenic cells for therapeutic purposes [38]. An ideal cell tracer agent must also show effective cellular labeling and low cytotoxicity. Although SPION-based T2/T2^*∗*^ contrast agents have advantages, including high degrees of sensitivity and biocompatibility, there are numerous potential applications for stem cell labeling and tracking by MRI in both preclinical and clinical settings. However, the superparamagnetic iron oxide nanoparticles provided dark signals that might be the result of obliteration in the surrounding anatomy and could be nonspecific concerning the source of the dark signal, which could be indicative of microbleeds, haemorrhages, the air/tissue interface, or released SPIONs internalized by macrophages [104]. To avoid this interference, an alternative bright-contrast cell tracking process using paramagnetic T1 contrast agents was applied in preclinical settings. Examples of T1 contrast agents identified as Gd-DTPA (Magnevist, FDA approved) and gadodiamide (Omniscan, FDA approved), as well as manganese porphyrin-based contrast agents, such as MnAMP, MnPNH_2_, and MnEtP, were applied in preclinical settings [104, 110–112]. Amongst T1 cell-labeling agents, there have been a plethora of approaches based on both gadolinium (Gd) and manganese. Clinically approved Gd chelating contrast agents are small, hydrophilic, and have cell-impermeable properties. In contrast, manganese porphyrins are naturally hydrophobic due to their larger aromatic macrocycles. Regarding their properties, these agents require high concentrations with or without transfection agents and use a long labeling time for cell uptake [113]. Here, we provided a new smart candidate T1 contrast agent, IronQ, for stem cell labeling. The preparation of IronQ involves a simple one-step synthesis from commercially available quercetin and iron (III) chloride. IronQ is a water-soluble agent and biologically safe. In the labeling process, IronQ are loaded up to 70 pg Fe per cell with a short incubation time of 24 h, and transfection agents are not required. In comparison to other paramagnetic T1 contrast agents, the main advantage of our novel smart candidate T1 contrast agent, IronQ, is its biological safety, as the main components are endogenous substances found in the human body and easily eliminated through physiological functions. Moreover, IronQ has almost no cytotoxicity in PBMCs and is suitable to use for stem cell tracking without negative effects on stem cell properties. We showed in this study that IronQ can induce the proliferation and differentiation of circulating angiogenic cells. Future studies will focus on optimizing the labeling conditions for several different types of therapeutic cells, such as hematopoietic stem cells, mesenchymal stem cells, and neural stem cells, for regenerative medicine. These analyses will be followed by *in vivo* studies to optimize the injection protocols for therapeutic cells in preclinical animal models of stem cell therapy. In the present study, we proposed IronQ as a positive T1 contrast agent with high potential for labeling the proangiogenic cells derived from human peripheral blood mononuclear cells and for tracking through MRI applications.

## 4. Conclusions

In the present study, we succeeded in synthesizing the iron (III)-quercetin complex IronQ with a stoichiometry ratio of 1 : 2 iron (III) and quercetin. The black powder IronQ product displayed the following physicochemical characteristics: a solid median size of 81 nm, a hydrodynamic diameter of 160.0 ± 2.4 nm in water, solubility in water, and a negative charge on the surface with a zeta potential of −24.53 ± 1.88 mV, showing paramagnetic behavior with a saturation magnetization of 0.155 emu/g at 1.0 T along with characteristic electronic light absorption peaks at wavelengths of 290 nm and 450–700 nm. We found that IronQ at 125 *μ*g/mL was safe and could be used in the long-term culturing (21 days) of PBMCs. Surprisingly, IronQ promoted the circulation of proangiogenic cell (CAC) growth in the conventional cell culture system without the addition of specific growth factors. IronQ can be uptaken into CACs depending on the dose and labeling time due to the advantages of the paramagnetic properties of IronQ, which allowed us to monitor the IronQ-labeled cells via MRI with the T1-weighted technique. IronQ displays paramagnetic T1-positive contrast agent properties accompanied by a longitudinal relaxivity of 2.29 mM^−1^s^−1^ for water and 3.70 mM^−1^s^−1^ for human plasma obtained at 25°C under a magnetic field of 1.5 T. The findings of this study clearly indicate that IronQ can enhance the T1 signal intensity on T1-weighted images in both water and plasma phantoms, as well as *in vitro* IronQ-labeled cells. Taken together, all evidence indicates that IronQ has excellent abilities as both an imaging probe for MRI and a stimulating agent favoring circulating angiogenic cell differentiation.

Therefore, we assert that IronQ could be useful for the labeling and tracking of autologous blood-derived endothelial circulation angiogenic cells and/or other stem cells in the application of vascularization and tissue regeneration via noninvasive magnetic resonance imaging in both preclinical and clinical settings.

## Figures and Tables

**Table 1:** FTIR information regarding the detected characteristic bands [60–62] of quercetin, the deprotonated quercetin, and IronQ.

Group assigned to the given band	Wavenumber (cm^−1^)		
	Quercetin	Deprotonated quercetin (pH 12)	IronQ
O–H stretching vibration of phenol	3386, 3263	3352, 3172	3353, 3195
C=O aryl ketonic stretch	1664 s	1637 w, 1739 w (shoulders)	1617 w, 1682 w, 1731 w (shoulders)
C=C aromatic ring stretching bands	1604 s, 1560 s, 1516 s, 1448 s, 1407 s	1592 s, 1504 w, 1436 m	1569 s, 1479 s, 1414 s
O–H bending of phenols	1376 s	1364 w	1382 w
C–H bonds in aromatic hydrocarbon bending (in-plane)	1315 s, 1128 s, 1088 s, 1012 s, 932 s	1308 w (shoulder), 1108 m, 1060 m, 1019 m, 950 w	1337 w, 1121 w, 1085 s 1024 w, 976 m, 955 w,
C–O stretching of aryl ether (C–O–C)	1260 s	1272 s	1259 s
C–O stretching in phenol	1196 s	—	—
C–CO–C stretching and bending in ketones	1164 s	1168 s	1162 s
C–H bending of aromatic hydrocarbons (out-of-plane)	884 s, 864 s, 844 s, 820 s, 795/792 w, 720 s, 676, 632 s, 599 s	890w, 830/808 w, 768 s, 678 w, 642 s, 588 m	910 w, 808 s, 739 w, 617 m
Fe–O stretching	—	—	470 s

*Note*. s = strong, m = moderate, w = weak.

## Data Availability

The data used to support the findings of this study are available from the corresponding author upon request.

## Supplementary Materials

Supplementary Materials Supplementary Materials: Figure S1: ^1^H NMR spectra for quercetin. Figure S2: ^1^H NMR spectra for deprotonated quercetin. Figure S3: ^1^H NMR spectra for IronQ.
