# Supplementary material for: Iron (III)-Quercetin Complex: Synthesis, Physicochemical Characterization, and MRI Cell Tracking toward Potential Applications in Regenerative Medicine
Source: Contrast Media Mol Imaging. 2020 Dec 29;2020:8877862. doi: 10.1155/2020/8877862 (PMC7785384; doi:10.1155/2020/8877862)
Supplement: Supplementary Materials — Figure S1: 1H NMR spectra for quercetin. Figure S2: 1H NMR spectra for deprotonated quercetin. Figure S3: 1H NMR spectra for IronQ. [file 8877862.f1.docx]

**Supplementary Materials**

**Iron(III)–Quercetin Complex: Synthesis, Physicochemical Characterization, and MRI Cell Tracking Toward Potential Applications in Regenerative Medicine**

Phakorn Papan^1,2^, Jiraporn Kantapan^1^, Padchanee Sangthong^2^, Puttinan Meepowpan^2^, Nathupakorn Dechsupa^1^*

^1^Research Unit of Molecular Imaging Probes, Department of Radiologic Technology, Faculty of Associated Medical Sciences, Chiang Mai University, Chiang Mai 50200, Thailand

^2^Department of Chemistry, Faculty of Science, Chiang Mai University, Chiang Mai 50200, Thailand

* Corresponding Author

Nathupakorn Dechsupa

Email: [nathupakorn.d@cmu.ac.th](mailto:nathupakorn.d@cmu.ac.th)

**Details of supporting information:**

Number of pages: 4

Number of supporting figures: 3


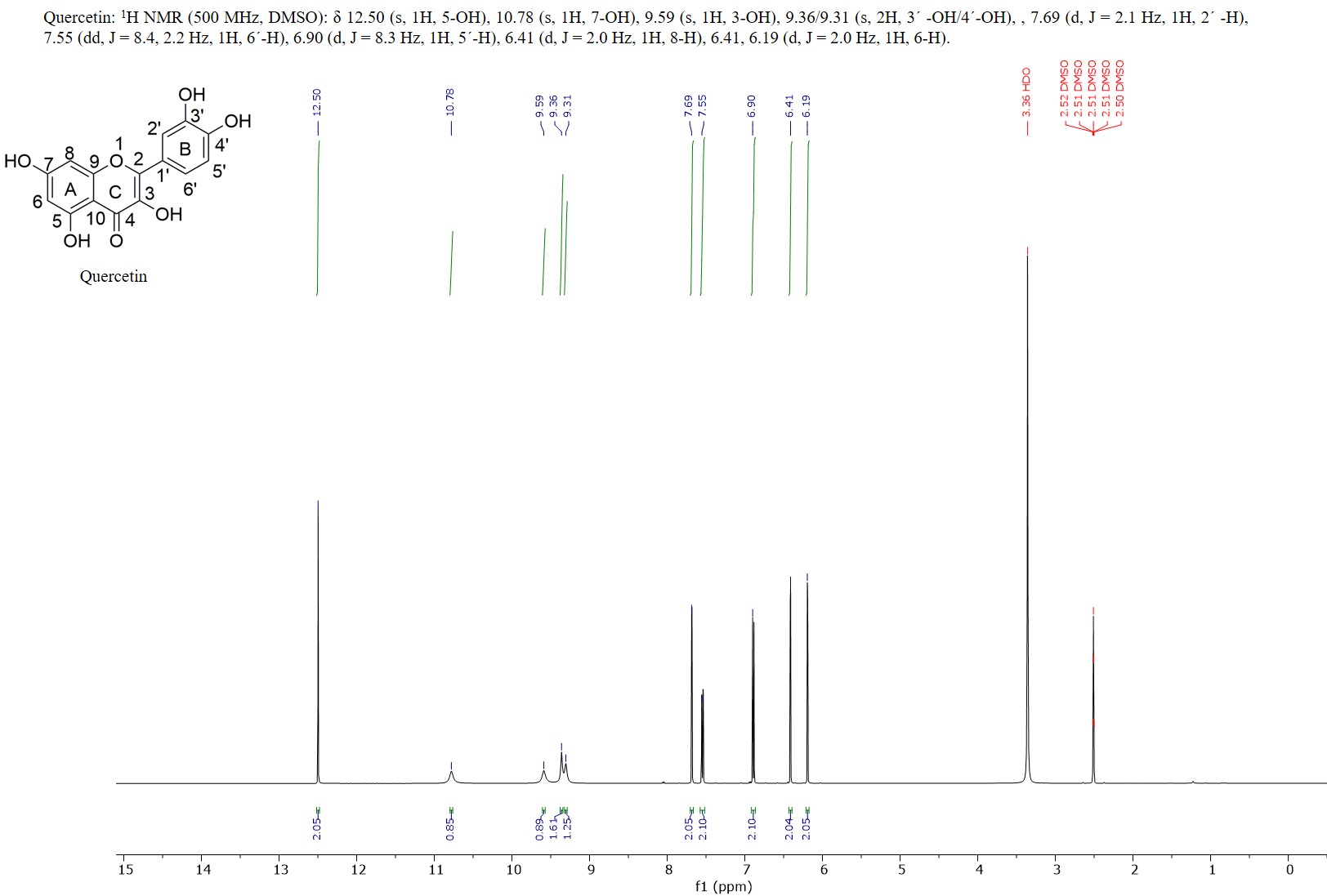


Figure S1: ^1^H-NMR (500 MHz, DMSO) of quercetin.

**
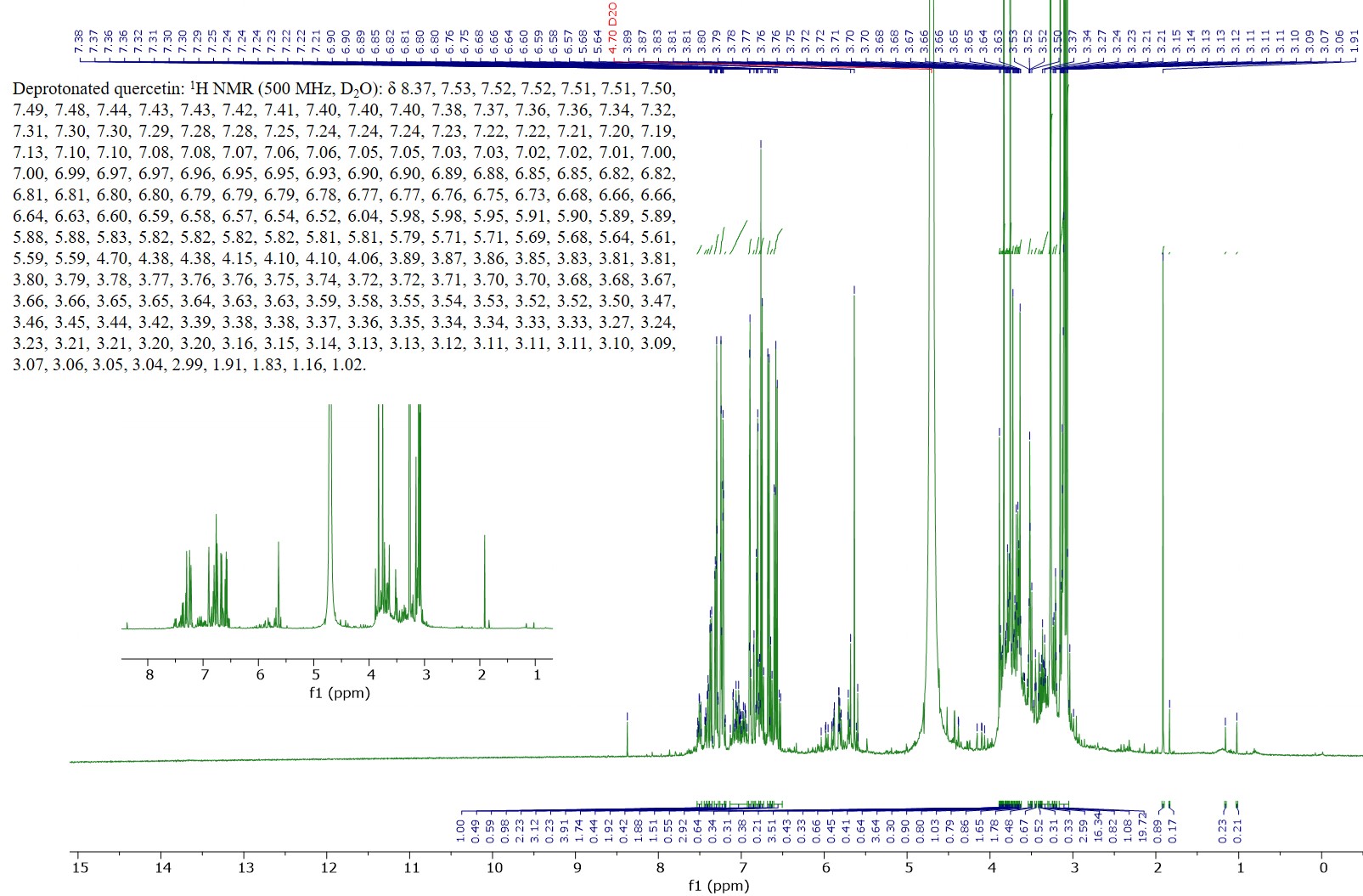
**

Figure S2: ^1^H-NMR (500 MHz, D_2_O) of deprotonated quercetin.

**
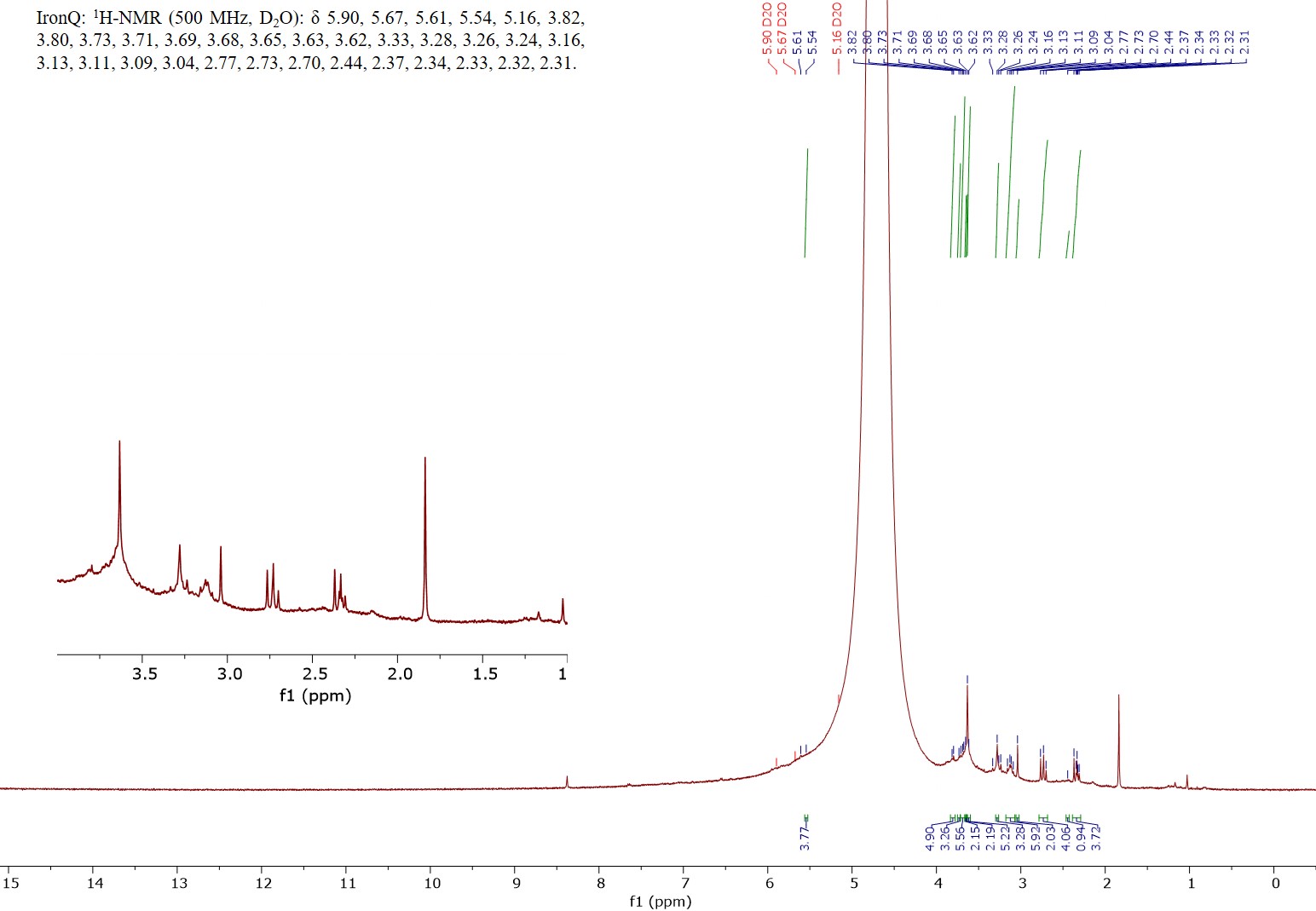
**

Figure S3: ^1^H-NMR (500 MHz, D_2_O) of IronQ.
